# GepLiver: an integrative liver expression atlas spanning developmental stages and liver disease phases

**DOI:** 10.1038/s41597-023-02257-1

**Published:** 2023-06-10

**Authors:** Ziteng Li, Hena Zhang, Qin Li, Wanjing Feng, Xiya Jia, Runye Zhou, Yi Huang, Yan Li, Zhixiang Hu, Xichun Hu, Xiaodong Zhu, Shenglin Huang

**Affiliations:** 1 Department of Medical Oncology, Fudan University Shanghai Cancer Center, and Shanghai Key Laboratory of Medical Epigenetics, International Co-laboratory of Medical Epigenetics and Metabolism, Institutes of Biomedical Sciences, Fudan University, Shanghai, 200032 China; 2 Department of Oncology, Shanghai Medical College, Fudan University, Shanghai, China

**Keywords:** Liver diseases, RNA sequencing

## Abstract

Chronic liver diseases usually developed through stepwise pathological transitions under the persistent risk factors. The molecular changes during liver transitions are pivotal to improve liver diagnostics and therapeutics yet still remain elusive. Cumulative large-scale liver transcriptomic studies have been revealing molecular landscape of various liver conditions at bulk and single-cell resolution, however, neither single experiment nor databases enabled thorough investigations of transcriptomic dynamics along the progression of liver diseases. Here we establish GepLiver, a longitudinal and multidimensional liver expression atlas integrating expression profiles of 2469 human bulk tissues, 492 mouse samples, 409,775 single cells from 347 human samples and 27 liver cell lines spanning 16 liver phenotypes with uniformed processing and annotating methods. Using GepLiver, we have demonstrated dynamic changes of gene expression, cell abundance and crosstalk harboring meaningful biological associations. GepLiver can be applied to explore the evolving expression patterns and transcriptomic features for genes and cell types respectively among liver phenotypes, assisting the investigation of liver transcriptomic dynamics and informing biomarkers and targets for liver diseases.

## Background & Summary

Liver is the largest solid organ in the body and plays a vital role in maintaining homeostasis with multidimensional functions. Serving as an essential hub for metabolic and immunological activities, liver could be vulnerable to various pathogenic factors including virus, alcohol, autoimmunity and metabolic disorders. These triggers contribute to stepwise pathological changes typically developing from repetitive liver damage and inflammation through fibrosis and cirrhosis potentially advancing to liver failure or malignant tumor^1,2^. Although histological transitions of liver diseases have been generally charted thanks to liver biopsies and surgeries, the dynamic and heterogeneous molecular changes during transitions remain poorly dissected, impeding the development of biomarkers and therapeutic targets for early prediction and tailored intervention of liver diseases.

Transcriptome, as the whole set of transcripts in a biospecimen, can demonstrate the overall molecular pattern of liver under a specific developmental stage or biological state^3^. High throughput sequencing-based methods have comprehensively revealed the genome-wide landscape of liver transcriptome and expanded the knowledge about liver homeostasis and pathogenesis across species^4–7^. The advent of single-cell RNA sequencing which measures gene expression of individual cells further revolutionized our understanding of liver biology through in-depth exploration of cellular heterogeneity and molecular perturbations at unprecedented resolution^8–11^. Shared and distinct features among liver phenotypes are being revealed by several transcriptomic studies. For instance, Yoon SH *et al*. examined bulk RNA-seq of HCC and paired premalignant lesions and demonstrated a depletion pattern of most immune cell types with Tregs and macrophages enriched on the contrary during HCC development^12^. Through single cell techniques, the identification of fetal-like PLVAP + endothelial cells and FOLR2 + macrophages in HCC also addressed the shared onco-fetal reprogramming of liver microenvironment between liver tumor and fetal liver^11^. However, a single experiment can hardly enable a thorough investigation of molecular dynamics during all stages of liver development and disease progression due to its limited sample size, liver phenotypes and mouse models. Moreover, despite multiple databases devoted to organize liver transcriptomic experiments for reuse, they either involved limited liver states and data modalities, such as Human Cell Atlas initiative (https://data.humancellatlas.org/explore/projects) focusing on single cell RNA-seq data of mostly healthy livers, or provided no access to data integration like Expression Atlas of EMBL-EBI^13^, which pressed the need for an effort to systematically integrate expression profiles across diverse models and liver conditions with uniformed processing and annotating methods.

**Fig. 1:**
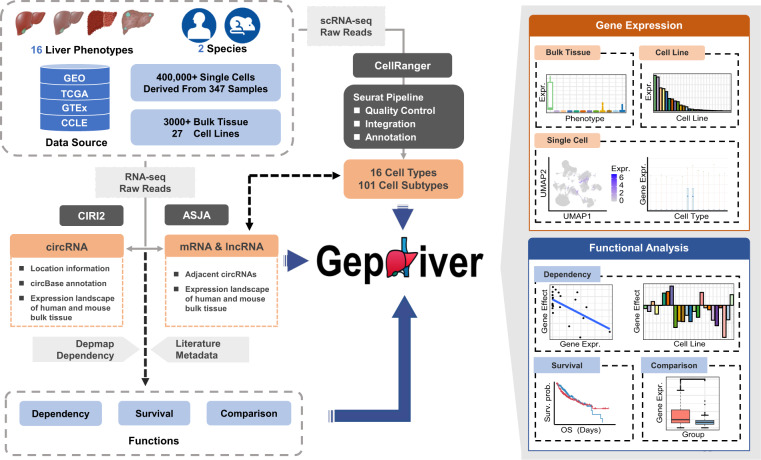
The overview of GepLiver workflow and main content. GepLiver curated RNA sequencing data of 2469 human bulk tissues, 492 mouse liver samples, 409,775 single cells from 347 human samples and 27 human liver cell lines in total covering 16 liver phenotypes and 2 species. RNA-seq raw reads were processed through the standardized pipeline of quality control, reads mapping and feature quantification using Assembling Splice Junctions Analysis (ASJA) and circRNA Identifier (CIRI2) algorithm whereas raw data of single cell RNA-seq were reanalyzed by CellRanger followed by downstream analysis of Seurat. Single cell datasets involved were harmonized into a liver reference map from which 16 cell types and 101 subtypes were finely identified. The expression landscape of normalized transcripts was further combined with gene dependency scores and literature metadata for functional analysis. GepLiver facilitates the visualization and direct comparison of gene expression among various liver phenotypes of bulk tissue, cell lines and single cells. The Analysis section including dependence, survival and comparison modules was provided for function explorations.

Towards this goal, we have established GepLiver which is a longitudinal and multidimensional liver expression atlas integrating RNA sequencing data of liver cells and tissue across the whole spectrum of liver developmental stages and diseases with unified processing pipeline (Fig. 1). The integrated data resource was deposited at figshare^14^ as well as the web-accessible GepLiver database (www.gepliver.org). Compiling both public resources and local cohorts, the first release of GepLiver compendium have encompassed 2469 human bulk tissues, 492 mouse liver samples, 409,775 single cells derived from 347 human samples and 27 human liver cell lines in total at present with phenotypes involving normal liver of all ages, hepatitis and cirrhosis of various causes, premalignant lesions as well as major liver tumor types. Straightforward comparisons among different liver phenotypes, mouse models and cell populations were facilitated for expression profiles of 45,860 mRNAs, 54,865 lncRNAs and 72,816 circRNAs. Transcriptomic dynamics was further associated with gene functions and clinicopathological information with the additional incorporation of gene dependency scores from Depmap project^15^ (https://depmap.org/portal/) and formalized metadata including survival outcomes. Additionally, the integrated single cell atlas generated 16 cell types and 101 subtypes of which fractions, biological signaling, differentiation states as well as intracellular interactions were evaluated and available for exploring dynamic changes at cellular level.

Applying GepLiver to explore liver transcriptomics dynamics along liver disease progression, we have identified several expression patterns enriched with distinct biological processes that were described to be dysregulated in liver disease, supporting the capability of GepLiver to uncover valid gene expression dynamics throughout liver transitions. Further validating the fidelity of the integrated single cell atlas to real biological states, the enrichment patterns of cell subtypes, biological processes and ligand-receptor interactions among liver phenotypes demonstrate agreement with literature on corresponding liver diseases.

GepLiver serves as a large-scale, integrated data resource and provided a user-friendly interface to investigate dynamic expression pattern during liver transition correlated with cell populations and clinical information, identify genes potentially associated with the progression of liver diseases, select appropriate mouse model for verification, and ultimately assist the whole developmental process of predictive biomarkers and therapeutic targets. Collectively, our atlas can be expected to shed novel light on liver pathophysiology as well as fuel both basic and clinical research for improved liver diagnostics and therapeutics.

## Methods

### Data acquisition

Keyword queries were combined with manual selection filtering for datasets included in GepLiver. For bulk liver tissue of human and mouse, we first retrieved RNA sequencing datasets as of February, 2022 from public resources (majorly GEO, ArrayExpress as supplement) searching terms associated with liver phenotypes, including “Fetal Liver”, “Fatty Liver”, “Alcohol AND Liver”, “Hepatitis”, “Liver AND (Fibrosis OR Cirrhosis)”, “Liver AND (Tumor OR Cancer)”, “(Hepatocellular Carcinoma) OR Hepatoma”, “Cholangiocarcinoma”, “(Biliary Tract) AND Cancer” and “Hepatoblastoma”. Studies derived from human liver tissue were next selected based on the following criteria: with downloadable raw reads (FastQ or Bam files); with more than 10 samples (except for fetal liver and alcoholic liver diseases due to less data available); with at least one related publication for reliable experiment protocols and patient metadata. Healthy livers of all ages from GTEx project and samples with HCC and ICC from TCGA initiative were also included into the compendium.

Mouse models conducted on C57BL/6 strain were prior to be considered to keep relatively consistent genetic backgrounds and for those studies with duplicated model designs, only experiments with larger sample size were kept.

Considering less accessibility of raw data of single cell RNA-seq data, we expanded the queries to GSA (Genome Sequencing Archive, https://ngdc.cncb.ac.cn/gsa/) in addition to GEO database. Studies with more samples, more abundant cell types and less stringent sorting strategies were prior to be included. And projects performed by the 10x genomics platform were selected to unite the preprocessing pipeline. Moreover, we included expression profiles of cancer cell lines with HCC and ICC collecting raw reads from CCLE project.

Ultimately, 35 datasets of human liver tissue, 1 dataset of human liver cancer cell lines, 17 datasets of mouse liver models as well as 17 human single cell studies were involved in GepLiver (Table 1, 2). This data compilation comprised RNA-seq experiments of 2469 human bulk tissue, 492 mouse liver samples, 409,755 single cells derived from 347 human samples as well as 27 human liver cell lines in total covering 16 liver conditions across the entire range of liver developmental stages and biological conditions (**File “Sample Descriptions”** deposited at figshare^14^).

**Table 1:** Summary of bulk RNA-seq datasets involved in GepLiver.

Dataset	Source	Reference	Selection	Layout
GepLiver-bulk-01	GTEx	GTEx^5^	PolyA	Paired
GepLiver-bulk-02	GSE114150	Xiao, S. *et al*.^44^	rRNA-d	Paired
GepLiver-bulk-03	GSE128102	Touboul, T. *et al*.^45^	rRNA-d, PolyA	Paired
GepLiver-bulk-04	GSE78569	ENCODE^46^	rRNA-d	Paired
GepLiver-bulk-05	GSE126848	Suppli, M. P. *et al*.^47^	PolyA	Single
GepLiver-bulk-06	GSE130970	Hoang, S. A. *et al*.^48^	PolyA	Paired
GepLiver-bulk-07	GSE135251	Govaere, O. *et al*.^7^	PolyA	Paired
GepLiver-bulk-08	GSE162694	Pantano, L. *et al*.^49^	rRNA-d	Single
GepLiver-bulk-09	GSE167523	Kozumi, K. *et al*.^50^	PolyA	Single
GepLiver-bulk-10	GSE142530	Massey, V. *et al*.^51^	rRNA-d	Paired
GepLiver-bulk-11	GSE143318	Hyun, J. *et al*.^52^	rRNA-d	Single
GepLiver-bulk-12	GSE155907^53^	N/A	rRNA-d	Paired
GepLiver-bulk-13	E-MTAB-6863	Ramnath, D. *et al*.^54^	rRNA-d	Single
GepLiver-bulk-14	GSE112221	Hlady, R. A. *et al*.^55^	PolyA	Paired
GepLiver-bulk-15	GSE144269	Candia, J. *et al*.^56^	rRNA-d	Single
GepLiver-bulk-16	GSE84346	Boldanova, T. *et al*.^57^	PolyA	Single
GepLiver-bulk-17	GSE94660	Yoo, S. *et al*.^58^	PolyA	Paired
GepLiver-bulk-19	TCGA	TCGA^4^	PolyA	Paired
GepLiver-bulk-20	GSE114564	Kim, S. S. *et al*.^59^	rRNA-d	Paired
GepLiver-bulk-21	GSE124535	Jiang, Y. *et al*.^42^	PolyA	Paired
GepLiver-bulk-22	GSE140462	Hall, Z. *et al*.^60^	rRNA-d	Single
GepLiver-bulk-23	GSE148355	Yoon, S. H. *et al*.^12^	rRNA-d	Paired
GepLiver-bulk-24	GSE77314	Liu, G. *et al*.^61^	PolyA	Paired
GepLiver-bulk-25	GSE77509	Yang, Y. *et al*.^62^	rRNA-d	Paired
GepLiver-bulk-26	TCGA	TCGA^63^	PolyA	Paired
GepLiver-bulk-27	GSE107943	Ahn, K. S. *et al*.^64^	PolyA	Paired
GepLiver-bulk-28	GSE119336^65^	N/A	PolyA	Paired
GepLiver-bulk-29	GSE162396	Kim, H. D. *et al*.^66^	PolyA	Single
GepLiver-bulk-30	GSE63420	Sia, D. *et al*.^67^	PolyA	Single
GepLiver-bulk-31	Fudan-ICC	Dong, L. *et al*.^68^	PolyA	Paired
GepLiver-bulk-32	GSE104766	Hooks, K. B. *et al*.^69^	PolyA	Paired
GepLiver-bulk-33	GSE133039	Carrillo-Reixach, J. *et al*.^70^	PolyA	Paired
GepLiver-bulk-34	GSE151347	Wagner, A. E. *et al*.^71^	PolyA	Paired
GepLiver-bulk-35	GSE81928	Valanejad, L. *et al*.^72^	PolyA	Paired
GepLiver-bulk-36	GSE89775	Ranganathan, S. *et al*.^73^	PolyA	Paired
GepLiver-bulk-38	CCLE	CCLE^74^	PolyA	Paired
GepLiver-bulk-39	GSE108348	Darbellay, F. *et al*.^75^	PolyA	Single
GepLiver-bulk-40	GSE109345	van Koppen, A. *et al*.^76^	PolyA	Single
GepLiver-bulk-41	GSE165752	Broadfield, L. A. *et al*.^77^	PolyA	Single
GepLiver-bulk-42	GSE162876	Loft, A. *et al*.^78^	PolyA	Paired
GepLiver-bulk-43	GSE166353	Sun, L. *et al*.^79^	rRNA-d	Paired
GepLiver-bulk-44	GSE48052	Lee, S. M. *et al*.^80^	PolyA	Single
GepLiver-bulk-45	GSE95424	Kan, F. *et al*.^81^	PolyA	Paired
GepLiver-bulk-46	GSE166868	Holland, C. H. *et al*.^82^	rRNA-d	Paired
GepLiver-bulk-47	GSE148379	Molina-Sánchez, P. *et al*.^6^	PolyA	Paired
GepLiver-bulk-48	GSE153077^83^	N/A	PolyA	Single
GepLiver-bulk-49	GSE90497	Shalapour, S. *et al*.^84^	PolyA	Single
GepLiver-bulk-50	GSE99010	Tsuchida, T. *et al*.^85^	PolyA	Paired
GepLiver-bulk-51	PRJNA488497	Dow, M. *et al*.^86^	PolyA	Single
GepLiver-bulk-52	GSE141511	Di-Luoffo, M. *et al*.^87^	rRNA-d	Paired
GepLiver-bulk-53	GSE150504	Cristinziano, G. *et al*.^88^	PolyA	Paired
GepLiver-bulk-54	GSE87578	Wang, H. *et al*.^89^	PolyA	Single
GepLiver-bulk-55	GSE156545	Wang, H. *et al*.^90^	PolyA	Paired

For each bulk RNA-seq dataset included in GepLiver, the data source, related publication, library selection method and layout were provided in the table. PolyA: PolyA-selected; rRNA-d: rRNA-depleted; Paired: Paired-end; Single: Single-end.

**Table 2:** Summary of single cell RNA-seq datasets involved in GepLiver.

Dataset	Source	Reference	Selection	Layout	Sorting
GepLiver-sc-01	GSE115469	MacParland, S. A. *et al*.^9^	PolyA	Paired	N/A
GepLiver-sc-02	CRA002443	Wang, X. *et al*.^39^	PolyA	Paired	N/A
GepLiver-sc-03	GSE159977	Pfister, D. *et al*.^91^	PolyA	Paired	CD45+
GepLiver-sc-04	GSE174748	Filliol, A. *et al*.^92^	PolyA	Paired	N/A
GepLiver-sc-05	GSE186328^93^	N/A	PolyA	Paired	N/A
GepLiver-sc-06	GSE192740	Guilliams, M. *et al*.^94^	PolyA	Paired	CD45+/−
GepLiver-sc-07	GSE217235	Woestemeier, A. *et al*.^95^	PolyA	Paired	CD45RA-CD4 + T
GepLiver-sc-08	GSE186343^96^	N/A	PolyA	Paired	N/A
GepLiver-sc-09	GSE200173	Koh, J. Y. *et al*.^37^	PolyA	Paired	CD45+
GepLiver-sc-10	GSE136103	Ramachandran, P *et al*.^10^	PolyA	Paired	CD45 +/−
GepLiver-sc-11	GSE168933	Buonomo, E. L. *et al*.^97^	PolyA	Paired	N/A
GepLiver-sc-12	GSE156625	Sharma, A. *et al*.^11^	PolyA	Paired	CD45 +/−
GepLiver-sc-13	SRP318499	Ho, D. W. *et al*.^98^	PolyA	Paired	N/A
GepLiver-sc-14	HRA001748	Xue, R. *et al*.^99^	PolyA	Paired	N/A
GepLiver-sc-15	GSE138709	Zhang, M. *et al*.^100^	PolyA	Paired	N/A
GepLiver-sc-16	GSE171899	Alvisi, G. *et al*.^101^	PolyA	Paired	CD45+/−
GepLiver-sc-17	GSE180665	Bondoc, A. *et al*.^102^	PolyA	Paired	N/A

For each single cell RNA-seq dataset included in GepLiver, the data source, related publication, library selection method, layout as well as the sorting strategy were provided in the table. sc: single cell; PolyA: PolyA-selected; Paired: Paired-end; Single: Single-end.

### RNA-seq raw data processing

Raw reads (Fastq or BAM file) of all bulk tissue and cell lines were retrieved and processed through the standardized pipeline of ASJA program^16^ (Assembling Splice Junctions Analysis, https://github.com/HuangLab-Fudan/ASJA) and CIRI2^16,17^ (circRNA Identifier v2.0.6, https://sourceforge.net/projects/ciri/files/CIRI2/). Briefly, we used FastQC software (v0.11.9, www.bioinformatics.babraham.ac.uk/projects/fastqc/) to assess the quality of fastq files and filtered out the low-quality reads with Trimmomatic (v0.33)^18^. Filtered reads were then aligned to hg38 or mm39 reference genomes via a two-pass mapping method provided by STAR software^19^ (v2.5.3a). Those reads mapping to mRNAs and long non-coding RNAs were quantified and normalized using featureCounts^20^ (v1.6.3) and further annotated to GENCODE V29, or VM28. For the identification and quantification of circRNAs, back-spliced junctions were extracted from STAR chimeric alignments through the Assembling Splice Junctions Analysis (ASJA) pipeline^16^. The identified circRNAs were further compared with the output of CIRI2^17^ (CircRNA Identifier) to reduce the false positive rate. The numbers of overlapped circRNAs were summarized to verify the data quality (**File “Mapping Statistics”** deposited at figshare^14^). Overlapped circRNAs identified by both ASJA and CIRI2 were then filtered for those expressed at least in 10 tissue samples with a sum of counts over 10 (For mouse species, the cutoff is at least in 3 samples and sum(counts) >3). Read counts are normalized using TPM (read counts scaled by gene length(kb) and sequencing depth) for mRNA and lncRNA and CPM (calculated by read counts/mapped reads*1 M) for circRNAs. For log-transformed expression value, we employed a base of 2 and a pseudo-count of 1. Each bulk RNA-seq dataset was processed from raw data separately as stated above. Popular methods reduce the batch effect either by changing the original gene expression values^21^ or only under specific analytic scenarios such as gene differential analysis provided by DEseq 2 R package. Therefore, we chose to Log2-normalize these bulk datasets and combined them into human or mouse merged expression matrix without any batch correction procedures to ensure the conservation of biological variance as possible.

### Single cell RNA-seq processing

#### Data processing using CellRanger

Raw reads (FASTQ or BAM files) of single cell RNA-seq datasets were downloaded from public resources. BAM files retrieved were converted into FASTQ files with bamtofastq (https://github.com/10XGenomics/bamtofastq) and then all FASTQ files were reanalyzed by cellranger count (CellRanger 6.0.0, 10X Genomics) through a pipeline of alignment, filtering and quantification with GRCh38 as human genome reference. The filtered feature-barcode matrices generated were used for downstream analysis.

#### Quality control

Downstream analysis for reprocessed expression matrices of human single cell RNA-seq was performed through Seurat R package^22^ (v4.1.0). Quality control procedure is separately employed to each sample with uniformed criteria. Briefly, cells expressed fewer than 300 genes and with a higher mitochondrial gene percent (taking the smaller one from 25% and 5th percentile of normal distribution modeling mitochondrial gene percent) were removed, as were genes expressed in less than 3 cells. Mitochondrial genes and ribosome genes were excluded from gene features to account for the variation in their percentages across samples. The filtered expression profile was processed through sequential steps of normalization, variable feature selection, dimension reduction, and clustering using Seurat functions (NormalizeData, FindVariableFeatures, ScaleData, RunPCA, FindNeighbours, FindClusters, RunUMAP).

Doublets were predicted using DoubletFinder R package^23^ (v2.0.3) for each sample derived from droplet-based protocol and removed before dataset integration. In brief, DoubletFinder randomly chose cell pairs and averaged their expression profile to produce artificial doublets. Through co-clustering of simulated doublets with real cells, a predefined proportion of real cells were predicted as doublets demonstrating proximity to artificial doublets in feature space with the first 20 PCs. nExp were calculated according to density of loading cells with reference to the Multiplet Rate Table provided in guidelines for 10X Single Cell Gene Expression (https://kb.10xgenomics.com/hc/en-us/categories/360000149952-Single-Cell-3-Gene-Expression). An optimal pK was identified with find.pK function. Other parameters were set as default. Ultimately, a total of 2,150,197 cells from 349 human samples were retained after quality control. Considering the extremely high memory consumption caused by million level cells, we subset 1500 cells per sample before data integration. Cell numbers before and after quality control as well as the subset quantities at the sample level were provided in **File “Single Cell Quality Control”** deposited at figshare^14^.

#### Data integration, clustering and annotation for liver atlas

The subset Seurat objects were merged into one object which was then normalized (NormalizeData, Lognormalized, scale.factor = 10,000). Most variable genes were found (FindVariableFeatures, n = 2000) and further scaled with the variation in feature counts (nFeature_RNA) regressed out. Principal component analysis was employed for linear dimension reduction with the first 50 PCs used to correct batch effect with Harmony^24^ (version 0.1.0, using theta = 1). The top 30 harmony dimensions were next provided for both non-supervised clustering (FindNeighbors and FindClusters, Louvain algorithm, clustering resolution = 0.2) and UMAP visualization of cell distance in low-dimension space. We defined xx clusters and assigned general cell identities referring to a list of canonical markers including ALB, EPCAM for epithelial cells; PECAM1, VWF and COL1A1 for stromal cells; CD14 and CD68 for myeloid lineage; CD3D, CD2 and GNLY for T and NK cells; CD19 and CD79A for B cells; IGHG1 for plasma cells; TPSAB1, TPSB2 for mast cells and HBA1 for erythroid cells.

#### Data integration, clustering and annotation for lineage subclusters

For detailed characterization of cell subtypes, we further subset cell lineages for clustering with higher resolution. Lymphoid (T cells and NK cells), myeloid (monocytes, macrophages, dendritic cells, neutrophils) and stromal cells (endothelial cells and fibroblasts) were separated from the integrated liver atlas to re-run the steps of normalization, variable feature selection, scaling as well as Harmony integration with different parameters used. In detail, for T cells and NK cells, 2000 variable features were selected and the first 20 PCs were used for clustering with resolution of 1; for myeloid cells, 1500 variable features were selected and the first 20 PCs were used for clustering with resolution of 1; for stromal cells, 1500 variable features were selected and the first 20 PCs were used for clustering with resolution of 0.8. As cells derived from fetal liver were clustered together in the broad cell clustering demonstrating too large variance from other phenotypes to be differentiated, we manually selected erythroid cells identified in the first-round annotation and re-clustered them to discern fetal-specific cell subtypes with 1000 variable features, 20 PCs and clustering resolution of 1.

For each lineage sub-clustering process, cell types were first assigned with well-acknowledged marker genes such as CD3D, CD4, CD8A, TRDC, TRGC1, TRGC2, GNLY, FCGR3A for characterizing CD4T, CD8T, Non-conventional T and NK-like cells, as well as CD14, S100A8, FCN1, CD163, CD1C, CSF3R for characterizing Monocyte, Macrophage, DC and Neutrophil. Next, markers of each cluster were further found using Seurat’s “FindMarkers” function with the default Wilcoxon Rank Sum test to discern subtle cell subpopulations. The threshold of log2FC and min.pct parameters were set to be 0.25 and 0.1 respectively. Only positive markers were obtained. Top 10 markers of each cluster were referred to curated literature and detailed annotations of all clusters were defined accordingly. Besides, suggested labels annotating cell functions and distribution were assigned for cell subtypes based on results of Single-cell Gene Signature Scoring and Cell abundance analysis described in the below sections (**File “Cell Annotations”** deposited at figshare^14^).

Epithelial cells were separated based on the expression of ALB, TTR, HNF4A for hepatocytes as well as KRT19, EPCAM, TM4SF4, FXYD2 for cholangiocytes. These cells were further annotated with the malignancy status according to inferred copy number variation.

At last, we characterized 16 cell types and 101 subtypes including normal and malignant subpopulation of Hepatocyte and Cholangiocyte as well as 7 CD4T, 6 CD8T, 7 Non-conventional T, 5 NK-like cell, 1 B cell, 1 Plasma cell, 3 Monocyte, 8 Macrophage, 9 Dendritic cell, 5 Neutrophil, 1 Mast cell, 10 Endothelial, 8 Fibroblast and 26 Fetal-derived subclusters.

#### Copy number variation analysis

We exploited inferCNV R package (version 1.10.1, inferCNV of the Trinity CTAT Project, https://github.com/broadinstitute/inferCNV) to recognize somatic large-scale chromosomal copy number aberrations by comparing gene expression level across each genome region with that of the reference cells. The CNV status was independently inferred for cells of each dataset with -cutoff 0.1 to avoid batch effect. All epithelial cells of malignant tumor samples were input for interrogation while epithelial cells and endothelial cells from normal or tumor-adjacent liver samples of the corresponding dataset were used as both references and spike-ins. For datasets with less than 200 epithelial cells or endothelial cells from liver samples with normal state, we selected these reference cells from GepLiver-single cell-01(GSE115469, 4 healthy livers) as surrogate.

#### Cell abundance analysis

Absolute and relative fractions were both computed for each cell type and subtype at atlas or phenotype level. Specifically, absolute fraction was calculated as the ratio of Num (one cell population) to Num of (cells of the broad liver atlas or specific liver phenotype). Due to the various cell sorting strategies employed by single cell datasets involved in our liver atlas, absolute fractions of cell types could be biased. To complement such bias, relative proportion of one cell type was obtained divided by quantities of corresponding cell lineage (Epithelial, Lymphoid, Myeloid, Stromal and Erythroid) among either the landscape or individual liver phenotype. Furthermore, the enrichment of liver phenotype in each cell type and subtype was evaluated through building a confusion matrix as follows:

**Table:** 

Num of (cell type i, phenotype j)	Num of (cell type i, the rest of phenotypes)
Num of (the rest of cell types, phenotype j)	Num of (the rest of cell types, the rest of phenotypes)

on which one-tailed hypergeometric test was performed to obtain the enrichment odds ratio and p value. The complete table containing results of abundance analysis was available in **File “Abundance of Cell Populations”** deposited at figshare^14^ and the Single Cell page of GepLiver website.

#### Single-cell gene signature scoring

We evaluated the single-cell activity scores of biological pathways and gene signatures with UCell^25^ R package (version 1.3.1) on the basis of relative rankings of involved genes for individual cells. Aiming to further interpret functional characteristics of distinct subpopulations from one cell type, we collected gene signatures, including M1/M2 polarization, Pro/anti-inflammatory cytokines, angiogenesis, phagocytosis and antigen-presentation for myeloid cells, memory, residency, cytotoxic and exhaustion markers for lymphoid cells as well as proliferative markers for all cells, from literature^26,27^ (**File “Gene Signatures for myeloid and T_NK”** deposited at figshare^14^) and then estimated their enrichment in corresponding cell clusters. Besides, 50 hallmark pathways recapitulating 50 non-redundant and representative biological processes were also retrieved from Msigdb database and evaluated for all individual cells to enable function characterization for any customized group of cells of interest. The enrichment scores of hallmark pathways were provided at **File “Single Cell Pathway Enrichment”** deposited at figshare^14^.

#### Cell differentiation and trajectory inference

Cell differentiation states of the integrated liver single cell atlas were respectively evaluated using CytoTRACE^28^ R package (version 0.3.3). CytoTRACE is a computational algorithm scoring relative developmental potential of single cells based on gene counts per cell indicating transcriptomic diversity. We performed CytoTRACE analysis for count matrices of 16 cell types separately. Considering potential batch effects, datasets with cells less than 100 were removed and then the function iCytoTRACE was applied with default parameters. The CytoTRACE scores range from 0 (relatively more differentiated) to 1(relatively less differentiated). Scores computed as above for total cells were provided in **File “CytoTRACE scores”** deposited at figshare^14^.

We performed cell trajectory inference for myeloid cells and fetal-derived subsets of the liver single cell atlas using Monocle R package (version 2.22.0). The top 300 marker genes identified by Seurat Findmarkers for each cell subtypes were used as genes for ordering. Differentiation trajectories were built with default parameters after dimension reduction and cell ordering.

#### Cell communication

We inferred intercellular communication network with CellPhoneDB^29^ (version 3.1.0) among 16 cell types identified with medium resolution. Integrating a curated ligand-receptor database, CellPhoneDB identified the enriched interactions based on expressions of ligand and receptor in source and target cell type respectively, followed by permutation tests for significance. In this study, the log2-normalized count matrix of the liver single cell atlas was first split into 13 chunks grouped by liver phenotypes. For phenotypes with cells over 50000, the expression matrix was downsampled to include 50000 cells using stratified sampling based on cell types. The significant interaction counts among cell types and cell type-specific interaction strengths for ligand-receptor pairs were then calculated using statistical analysis function of CellphoneDB with default parameters. Intercellular interactions among cell types were visualized for 13 liver phenotypes separately which could be accessed at Cell Communication subsection on the Single Cell Page of GepLiver website (www.gepliver.org/#/explore).

### Cell type deconvolution in human bulk sequencing data

For the integration of bulk and single cell RNA-seq datasets, a feature matrix of 16 cell types identified from our atlas was generated and then applied to deconvolute the corresponding cell proportions in RNA-seq expression profiles of 2469 human bulk samples hosted in GepLiver via the CIBERSORTx^30^ website (cibersortx.stanford.edu/runcibersortx.php) with 200 permutations and no quantile normalization. The decerned cell fractions for human bulk samples were available at **File “Cell Type Deconvolution for Human Bulk”** deposited at figshare^14^ as well as Human Abundance-Bulk subsection in Single Cell page of GepLiver website.

### Functional analysis

#### Gene dependency

GepLiver incorporated the Chronos dependency score from Depmap^15^ project (https://depmap.org/portal/download/all/, Public 22Q1) to suggest the functional role of gene of interest over the viability of liver cancer cell lines. Briefly, this dependency score is derived from CRISPR gene knockout assay with a lower score indicating that the selected gene is more likely to affect the viability or proliferation of the specific cell line. Genes with scores of 0 means non-essential for the given cell line. A bar plot of gene dependency scores across cell lines and a scatter plot showing the correlation between dependency scores and corresponding gene expression values were visualized in Dependency module of GepLiver Analysis section (www.GepLiver.org/#/analysis).

#### Survival analysis

Several types of survival data, including Overall Survival (OS), Disease Specific Survival (DSS), Disease Free Survival (DFS) and Progress Free Survival (PFS) were acquired from patient metadata of the included cohorts. To explore the prognostic significance of the given gene, both the log-rank test and the univariate Cox proportional hazards regression analysis were performed for survival analysis.

### Metadata standardization

GepLiver collected metadata of each dataset provided by GEO portal using getGEO function of GEOquery R package and combined them with supplementary information from related publications. Suffer from incomplete records and various classification methods. we managed to standardize those important fields including project ID, sample type, age, sex, risk factors, inflammation grade, fibrosis stage as well as clinicopathological parameters of liver tumor. The age field was segmented into 6 groups: <0 (fetal); 0–1 y; 1y–17y; 18y–49y; 50y–69y; > = 70 y. Multiple grading systems for liver inflammation and fibrosis were used in GepLiver datasets: NAS score^31^ and METAVIR^32^ activity grade were adopted for inflammation evaluation whereas METAVIR^32^ and Ishak system^33^ were all applied to fibrosis staging. To unify these standards, we interrogated their pathological measurements and then reclassified them into a uniformed four-stage grading system. Specifically, the degree of liver inflammation was determined as “None”, “Mild”, “Medium” and “Severe” merging from NAS_0 and METAVIR A0, NAS_1-3 and METAVIR A1, NAS_4-6 and METAVIR A2 as well as NAS_7-8 and METAVIR A3 respectively. The extent of liver fibrosis was graded into “None”, “Low”, “High” and “Cirrhosis” which respectively consisted of METAVIR F0 and Ishak stage0, METAVIR F1-2 and Ishak stage1-2, METAVIR F3 and Ishak stage3-5 as well as METAVIR F4 and Ishak stage6. Regarding clinical information of liver cancer, tumor stage, grade, size and survival data were provided if available. The metadata harmonization could further facilitate the identification and validation of biomarkers and targets across studies with higher statistical power.

### Modeling of transcriptomic dynamics in bulk RNA-seq

Gene expression patterns during liver transitions were modeled and clustered using STEM software (Short Time-series Expression Miner, v1.3.13)^34^ which was designed for the temporal analysis of gene expression profiles specifically with short time series. Briefly, expression profiles of two sets of liver phenotypes were chosen from GepLiver repository according to typical courses of virus-related HCC and non-alcoholic steatohepatitis (NASH), respectively. Log2 fold changes, as input of STEM, were calculated with limma R package making pairwise contrasts between each phenotype and normal liver samples. Transcriptomic dynamics across liver disease phases were modeled with “Normalize data” selected and genes with similar expression pattern were clustered using STEM clustering method by default. Maximum unit change between two consecutive time points was set as 10 to capture extreme expression changes during malignant transformation.

### Pathway over-representation analysis

Gene clusters were functionally annotated with pathway over-expression analysis provided by clusterProfiler R package (v4.4.4)^35^. HALLMARK, KEGG, REACTOME and BIOCARTA gene sets were acquired from msigdbr package (v7.5.1) and enriched with enricher function whereas over-expression of GO-BP gene sets was analyzed with enrichGO function.

## Data Records

The data at figshare^14^ represents a static copy of GepLiver web resource, reviewed in 2023.

The integrated data resource, including the annotation files, processed expression matrices and metadata, was publicly available at both figshare^14^ and “Download” page of GepLiver website (www.GepLiver.org/#/download). GepLiver comprehensively curated RNA sequencing data from 70 datasets of public resources (Tables 1, 2). The exploration of gene expression, cell locations and biological functions were also facilitated at GepLiver web interface.

File “Transcript Annotation” contained basic gene information and summary statistics for all transcripts covering 45,860 mRNAs, 54,865 lncRNAs and 72,816 circRNAs derived from human and mouse bulk tissue RNA-seq data. For mRNAs and lncRNAs, basic annotations including gene symbol, ensemble ID, species, gene type and numbers of related circRNA were provided while for circRNAs, chromosome locations, circBase ID and host gene information were annotated. Besides, statistics describing transcript level were also presented: average expression value (median value for mRNA and lncRNA, mean value for circRNA) and frequency of expressed samples for each gene within every liver phenotype have been calculated for all gene features.

Two Files, “Human Bulk Expression Matrix and Metadata” and “Mouse Bulk Expression Matrix and Metadata”, comprised TPM normalized counts and corresponding metadata for 35 datasets of human bulk tissue and 17 experiments of mouse liver samples, respectively. The “Run” column denoted the sample identifiers which were designated as the original sample ids for GTEx and TCGA project and sequencing run ids for samples from GEO repository. For human metadata, the “Treatment” column illustrated what kind of regimen patients were treated with before sample collection with those treatment-naïve designated as “None”. For mouse metadata, “Model_show” column contained mouse model types with recapitulated liver phenotypes annotated in the brackets. Time span for inducing corresponding disease model was provided in “Duration” column if available.

File “The integrated GepLiver single cell atlas” was the integrated scRNA-seq data provided as a Seurat object whereas standardized sample information was provided as File “Metadata for single cell atlas”. Embeddings of dimension reduction and uniformed cell type annotations were contained in the Seurat object to reproduce the liver single cell atlas of GepLiver.

File “Sample Descriptions” served as sample descriptions of 70 datasets involved in GepLiver, containing characteristics of samples including species, sample type, sample size, cell number, liver phenotype, risk factor and mouse model. The risk factor column describes etiologies for human liver diseases and intervention protocols for mouse models.

File “Mapping Statistics” provided the mapping statistics and circRNA numbers summarized for human and mouse bulk samples involved in GepLiver respectively in two sheets. Statistics provided for each dataset were calculated as the median value of corresponding parameters.

File “Single Cell Quality Control” contained the statistics for quality control process of the integrated single cell atlas. Cell quantities of the original sample, after uniformed quality control and after downsampling process were provided as columns of Orig_Num, QC_Num and Subset_Num respectively. The other four columns denoted the median of features including nCount_RNA, nFeature_RNA, mitochondrial percentage and ribosome percentage correspondingly.

File “Abundance of Cell Populations” comprised abundance analysis results for cell types and subtypes among individual liver phenotypes of integrated single cell atlas. Cell types and subtypes were dichotomized by “Medium” or “High” according to the clustering resolution column. Absolute fractions were computed against total cells whereas relative ones were calculated against corresponding lineage cells. OR > 1 and p < 0.05 denoted enrichment of the cell type in a specific phenotype.

File “Gene Signatures for myeloid and T_NK” provided gene signatures curated from literature to evaluate functions of myeloid and T/NK subclusters.

File “CytoTRACE scores” contained CytoTRACE scores calculated for total cells of GepLiver single cell atlas. To be noted, scores of cells from different cell types were incomparable since cell types were separated before evaluated to be developmentally meaningful.

File “Cell Annotations” supplied feature genes and suggested labels for cell types and subtypes of GepLiver single cell atlas. Feature genes were top10 most expressed genes identified with highest log2FC and percentage of expressed cells whereas suggested labels were assigned referring to marker expression and functional analysis.

File “Cell Type Deconvolution for Human Bulk” comprised deconvoluted cell fractions for human bulk samples involved in GepLiver based on feature matrix computed from integrated single cell data. Mean fractions of samples were calculated by liver phenotype for each cell type while Rela_fraction denotes the percentage of cell types divided by that of corresponding lineage. LogFC and p value were calculated compared with normal phenotype. P values were adjusted by Benjamini-Hochberg method.

File “Single Cell Pathway Enrichment” provided enrichment scores of 50 hallmark pathways from MsigDB database evaluated for total cells using UCell R package.

File “UMAP plots split by dataset and sample” supplied the comparison of UMAP plots at dataset or sample level colored by major cell types.

File “CellRanger gene-barcode matrix for single-cell datasets involved” contained three standardized output files generated by CellRanger which are features.tsv.gz, matrix.mtx.gz and barcodes.tsv.gz for all samples included in single cell atlas.

File “Liver Cancer Cell Line Expression Matrix” contained TPM normalized counts reanalyzed for 27 human liver cancer cell lines of CCLE project. The cell line identifiers were made up of cell line name and tumor primary site of LIVER or BILARY_TRACT.

File “Custom R Scripts” contained customed code used for data generation, processing and validation.

The resting two files were provided for suggesting functional significance for gene features. File “Liver Cancer Cell Line Dependency Score” collected Chronos dependency scores of 17081 genes from Depmap project (https://depmap.org/portal/download/all/, Public 22Q1) for 24 human liver cancer cell lines of CCLE. File “Survival Data” comprised patient outcomes of 4 HCC cohorts and 2 ICC cohorts.

## Technical Validation

### Quality control of RNA-seq processing

To inspect the sequencing quality of RNA-seq data included, the mapping statistics, including average mapped lengths and uniquely mapped reads have been summarized for all datasets (except for TCGA due to the retrieval of BAM files) with a median mapping ratio of 91.0% for human bulk RNA-seq and 82.3% for mouse RNA-seq data (**File “Mapping Statistics” deposited at figshare**^14^). Numbers of **c**ircRNAs recognized by both ASJA and CIRI2 algorithms for involved bulk RNA-seq datasets were also estimated to ensure the selection of circRNAs from datasets of higher quality for downstream analysis. For datasets ultimately included for circRNA analysis, a median of 5836 circRNAs were detected for human experiments and 2112 for mouse studies (**File “Mapping Statistics” deposited at figshare**^14^).

### Interrogating single cell integration efficiency

**Fig. 2:**
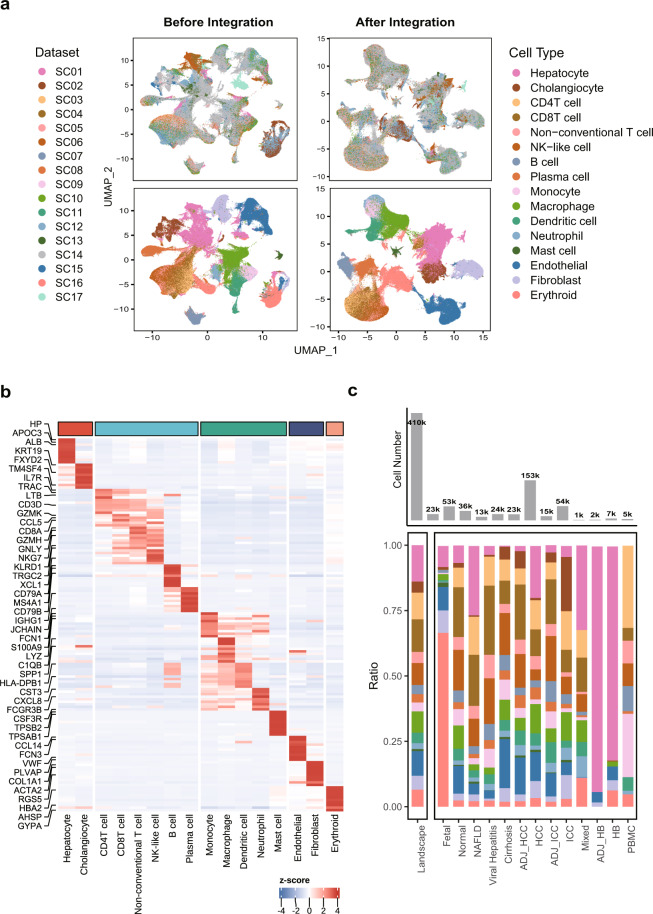
Validating the Harmony integration and cell type annotation for the landscape of single cell liver atlas. (**a**) The comparison of UMAP plot before and after dataset integration with the upper panel colored by datasets and the lower colored by 16 cell types; (**b**) The expression heatmap of top10 most expressed marker genes identified for cell types under the landscape with selected features labeled; (**c**) The comparison of absolute fractions of 16 major cell types among landscape and 13 liver phenotypes with cell number statistics demonstrated in the top bar plot; NAFLD, non-alcoholic fatty liver disease; ADJ, adjacent tissue; HCC, hepatocelluar carcinoma; ICC, intrahepatic cholangiocarcinoma; Mixed, mixed hepato-cholangiocellular carcinoma; HB, hepatoblastoma.

**Fig. 3:**
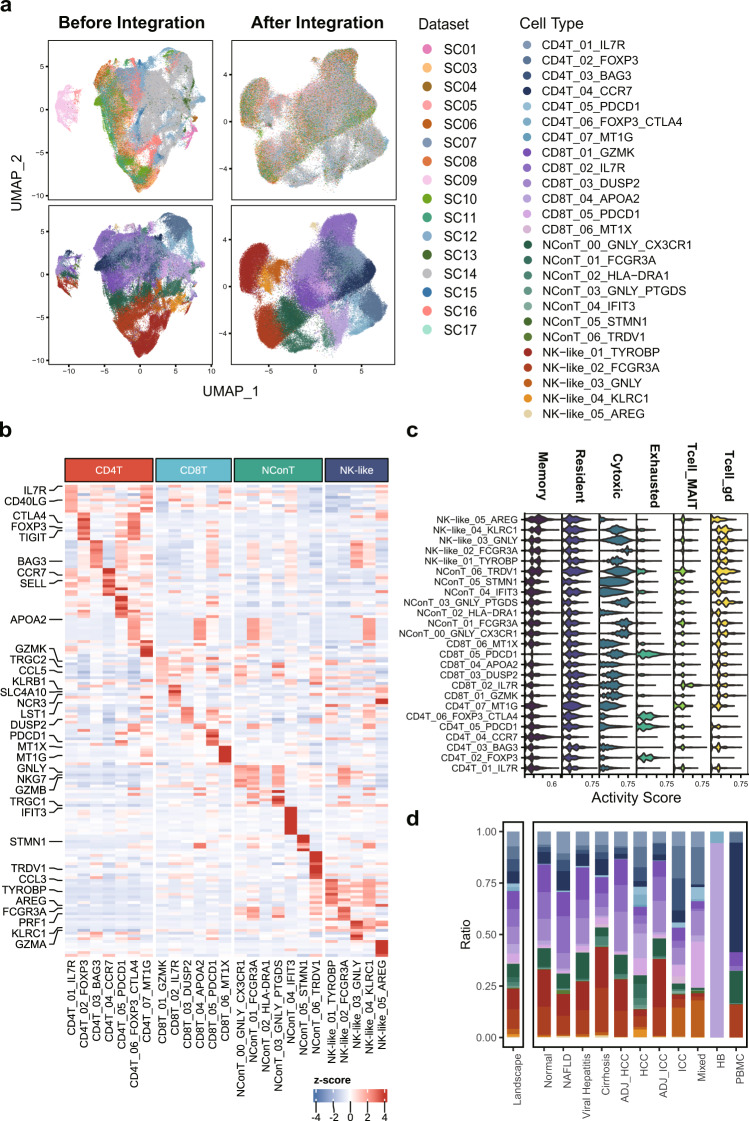
Validating the Harmony integration and cell subtype characterization for T/NK subsets. (**a**) The comparison of UMAP plots before and after dataset integration with the upper panel colored by datasets and the lower colored by 25 T/NK subpopulations; (**b**) The expression heatmap of top10 most expressed marker genes for cell subtypes identified among T/NK subsets with selected features labeled; (**c**) The enrichment scores of lymphoid signatures indicating functions (Memory, Resident, Cytotoxic and Exhausted markers) and cell identities (MAIT and gamma-delta T cell markers) computed for T/NK subtypes; (**d**) The comparison of cell subtype fractions relative to T/NK cells among landscape and 12 liver phenotypes (except Fetal). NConT, non-conventional T cell.

**Fig. 4:**
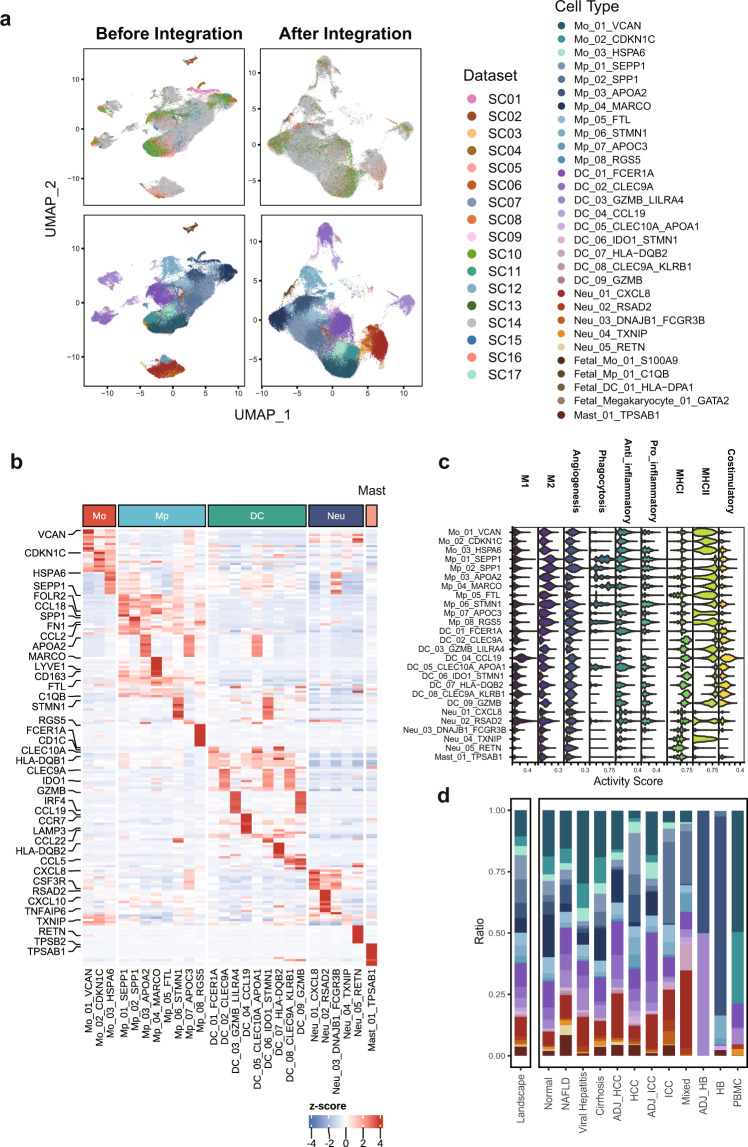
Validating the Harmony integration and cell subtype characterization for myeloid subclusters. (**a**) The comparison of UMAP plots before and after dataset integration with the upper panel colored by datasets and the lower colored by myeloid subpopulations (subtypes with less than 100 cells were not displayed); (**b**) The expression heatmap of top10 most expressed marker genes for cell subtypes identified among myeloid subsets with selected features labeled; (**c**) The enrichment scores of myeloid features computed for myeloid subtypes indicating functional heterogeneities; (**d**) The comparison of myeloid subtype fractions computed against the myeloid lineage among landscape and 13 liver phenotypes (fetal-derived subtypes were not included).

**Fig. 5:**
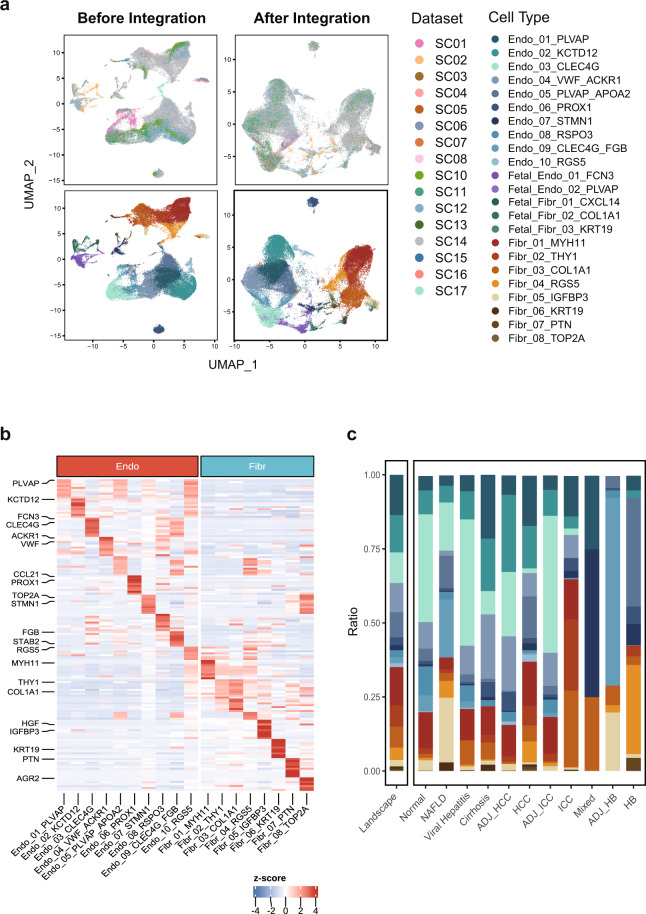
Validating the Harmony integration and cell subtype characterization for stromal subclusters. (**a**) The comparison of UMAP plots before and after dataset integration with the upper panel colored by datasets and the lower colored by stromal subpopulations; (**b**) The expression pattern of top10 most expressed marker genes for cell subtypes identified among stromal subsets with selected features labeled; (**c**) The comparison of stromal subtype fractions computed against the stromal lineage among landscape and 13 liver phenotypes (fetal-derived subtypes were not included).

**Fig. 6:**
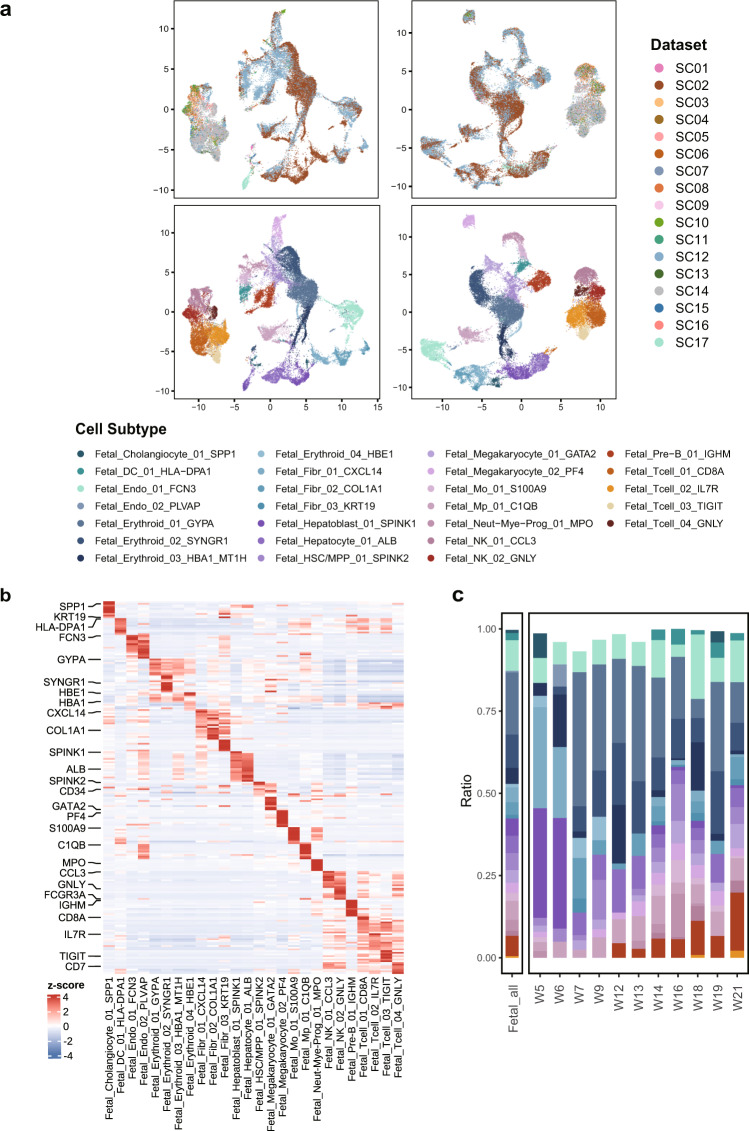
Validating the Harmony integration and cell subtype characterization for fetal-derived subclusters. (**a**) The comparison of UMAP plots before and after dataset integration with the upper panel colored by datasets and the lower colored by fetal-derived subpopulations; (**b**) The expression pattern of top10 most expressed marker genes for fetal subtypes identified with selected features labeled; (**c**) The comparison of fetal subtype fractions computed against all fetal-derived cells across liver developmental stages ranging from 5 to 21 post conception weeks; HSC/MPP, hematopoietic stem cell and multipotent progenitor.

Considering potential batch effects attributable to tissue quality, different protocols, sequencing technologies, cell recovery and sorting methods^36^, we integrated single cell datasets involved in GepLiver into a harmonized expression reference map using Harmony algorithm. To validate the integration performance, we plotted UMAP dimension reduction plots for atlas landscape grouped by datasets or cell types defined by canonical markers. As shown in Fig. 2a, cells, colored by experiments, were originally separated by both datasets and cell types with dataset-specific clusters displaying evident batch effects. After Harmony integration, populations from different datasets, such as hepatocytes from SC17 sequenced with single nucleus, were well mixed with corresponding cell types, validating both the mixing and accuracy of integration procedure. Similarly, we also compared the clustering patterns before and after integration process for lymphoid (Fig. 3a), myeloid (Fig. 4a), stromal (Fig. 5a) and fetal-derived subclusters (Fig. 6a), indicating satisfactory performance of integration among lineage subpopulations. For instance, CD45 + liver sinusoidal mononuclear cells, collected from healthy donors and patients with HBV-associated chronic liver disease from SC09^37^ (GSE200173), were ordered in a study-specific manner way from other lymphoid cells possibly due to liver perfusion and cell sorting process. Such dataset variance was harmonized after Harmony integration with cell subgroups merged well into clusters explained by biological differences.

### Validating the characterization of fine-grained cell subtypes from both expressional and functional aspect

The integrative liver single cell atlas generated 101 cell subpopulations originating from 16 major cell types. As these subclusters were discerned by non-supervised clustering algorithm with manually chosen parameters, we tried to interrogate whether cell subtypes with distinct biological characteristics were finely distinguished.

First, we evaluated the expression pattern of top 10 most expressed marker genes identified for 16 cell types under the atlas landscape (Fig. 2b) as well as for 101 subtypes under corresponding lineages (**b plots of** Figs. 3–6). Activity scores of functional signatures were additionally estimated for lymphoid (Fig. 3c) and myeloid subclusters (Fig. 4c). Well-acknowledged lineage markers demonstrated significantly cell type specific expression patterns as shown in Fig. 2b, indicating the well distinguishment of major cell types from each other.

Intrahepatic T/NK cells are a heterogeneous group of immune cells with highly complex functional variances. Among 25 T/NK subtypes, it is notable that CD4T and CD8T were distinctly separated from nonconventional T and NK-like populations both in UMAP plot (Fig. 3a) and the expression pattern of NK-like features (GNLY, NKG7, FCGR3A) (Fig. 3b). Subgroups among these four major types were also functionally interpretable. For instance, regulatory T cells and naïve/central memory T cells were both clearly identified from CD4 + T groups by the exclusive expression of FOXP3 and homing receptors SELL and CCR7, agreeing with the respective enrichment of exhaustion and memory signatures evaluated by UCell (Fig. 3c). Besides, CD8_02_IL7R featured cytokine secretion and the elevated activity of Mucosa-associated invariant T (MAIT) cell signature whereas two clusters of nonconventional T cells, NConT_03_GNLY_PTGDS and NConT_06_TRDV2, held significantly higher cytotoxic functions and gamma-delta features, indicating corresponding cell identities. These interrogations supported that distinct functional subgroups of intrahepatic T/NK cells were finely distinguished among our integrated single cell atlas.

For myeloid cell subsets, macrophages were dichotomously clustered into either angiogenic or phagocytic functional phenotype (Fig. 4c) whereas tissue-resident Kupffer cells were also clearly characterized with exclusive high levels of MARCO and LYVE1 (Fig. 4b), both agreeing with previous scRNA-seq findings over pan-cancer myeloid cells^26^. DC_03 and DC_09 featuring plasmacytoid DC marker gene LILRA4 consistently showed specialized expression of GZMB (Fig. 4b) whereas other DC subclusters instead demonstrated significant enrichment of MHCII pathway (Fig. 4c), indicating that conventional and plasmacytoid DC groups were distinctly separated in our integrated data.

Liver stromal cells, including endothelial cells and fibroblasts, manifest significant transcriptomic heterogeneity and functional zonation across the liver lobule due to compartmentalized vasculature^2,38^. Vascular, venous, lymphatic as well as liver sinusoid endothelial cells were definitely partitioned in our data respectively featuring the expression of VWF, RSPO3, PROX1 as well as CLEC4G marker genes (Fig. 5b). Four main liver mesenchymal cell types, vascular smooth muscle cells (VSMCs), Hepatic stellate cells (HSCs), Mesothelial cells as well as scar-associated mesenchymal cells reported enriched in liver cirrhosis were clearly delineated from our atlas exhibiting specialized expression of MYH11, RGS5, KRT19 and COL1A1 marker genes respectively (Fig. 5b).

Four cell lineage families, erythroid (hematopoietic stem cell and multipotent progenitor (HSC/MPP) and erythroid groups), non-erythroid hematopoietic (megakaryocytes, myeloid and lymphoid cells), endoderm-derived (hepatoblasts, hepatocytes and cholangiocytes) and mesoderm-derived non-hematopoietic lineages (endothelial cells and fibroblasts) were all distinctly characterized in the fetal subpopulations of liver single cell atlas with a more fine-grained annotation resolution compared to two datasets included in our atlas^11,39^ (Fig. 6b).

Collectively, these results validated the preservation of functional specialization of cell subtypes identified from GepLiver single cell data resource and supported that these distinct clusters represented biological variances rather than artifacts of batch.

### Conservation of differentiation trajectory in Myeloid cells and Fetal-derived clusters

We assumed that cell differentiation trajectories were preserved in our integrated liver atlas. Two cell types with known developmental relationships, myeloid cells (monocytes, macrophages and dendritic cells) and fetal-derived cells (erythroid and hematopoietic cell families) were selected to verify this assumption. We applied both CytoTRACE and Monocle to infer cell differentiation potential and developmental ordering respectively.

**Fig. 7:**
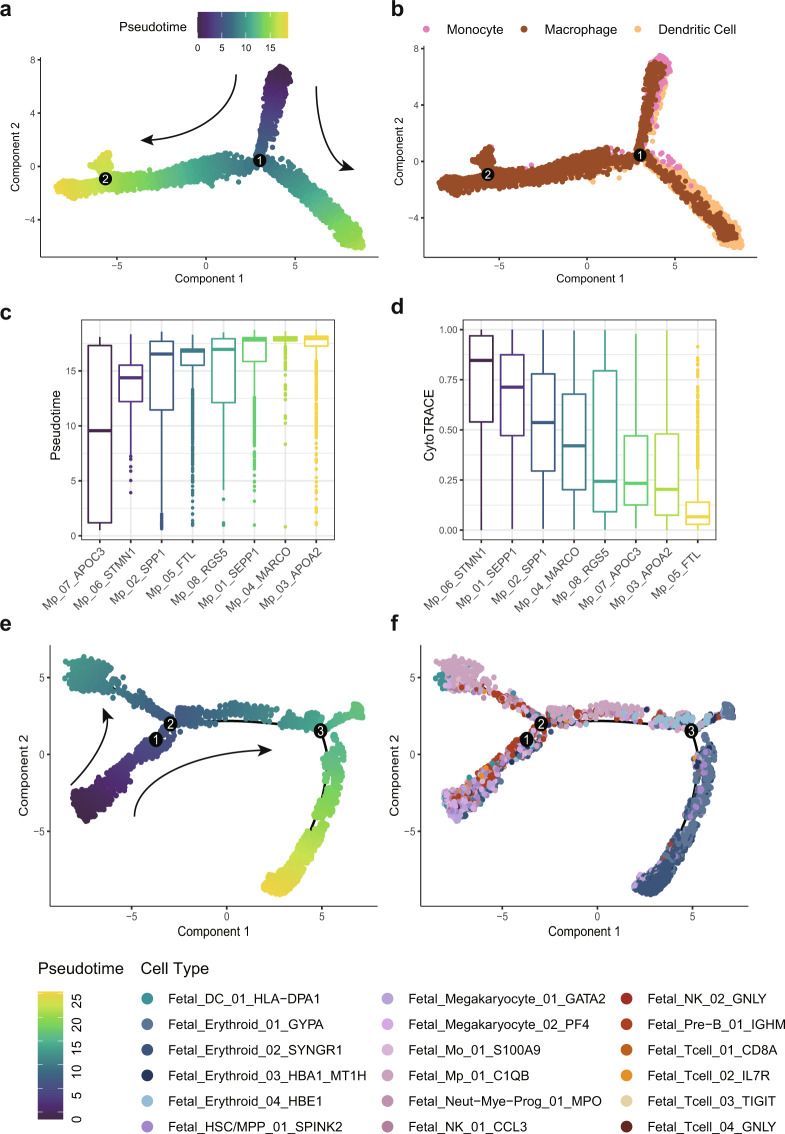
Interrogating the conservation of differentiation trajectories in myeloid cells and fetal-derived clusters. (**a**) The cell trajectories inferred for myeloid cells (monocytes, macrophages and dendritic cells) colored by pseudotime with differentiation direction labeled with arrows; (**b**) The cell trajectories inferred for myeloid cells (monocytes, macrophages and dendritic cells) colored by cell types; (**c**) The comparison of pseudotime among macrophage subtypes ordered from less differentiated (lower value) to more differentiated (higher value); (**d**) The comparison of CytoTRACE score among macrophage subtypes ordered from less differentiated (0) to more differentiated (1); (**e**) The cell trajectories inferred for fetal erythroid and hematopoietic cells colored by pseudotime with differentiation direction labeled with arrows; (**f**) The cell trajectories inferred for fetal erythroid and hematopoietic cells colored by cell subclusters.

As shown in Fig. 7a,b, cell trajectories built among three types of myeloid cells displayed that the macrophages and dendritic cells originated from different developing direction of monocytes, agreeing with the known developmental relationship of myeloid cells. Further the comparison of both pseudotime (Fig. 7c) and CytoTRACE score (Fig. 7d) among 9 macrophage subtypes suggested the less differentiated states, relative to liver resident Kupffer cell (Mp_04_MARCO), for cycling macrophage (Mp_06_STMN1) and two disease-associated macrophage subsets (Mp_01_SEPP1, Mp_02_SPP1) reported by studies involved in GepLiver. Consistently, Mp_01_SEPP1 featured higher levels of FOLR2 (Fig. 3b) also identified as a marker of fetal-like macrophage^11^.

For Fetal-derived subsets, monocle analysis suggested differentiation trajectory originating from HSC/MPP as expected into either lymphoid and myeloid lineage cells or megakaryocyte and erythroid cells (Fig. 7e-f), which was consistent with results identified by SC02 dataset^39^ (CRA002443). These results verified that cell trajectories were conserved and able to be recovered after integration of multiple datasets.

### Validity of cell abundance changes across liver phenotypes after integration

As single cell samples of liver phenotypes were sourced from different studies and utilized varying cell dissociation and sorting strategies, the resulting cell-type composition may be possibly biased after data integration. Considering that cells were also re-clustered and reannotated, it is necessary to evaluate whether the typical cell abundance change in the original studies can still be retained and accurately represented after integration. To address these concerns, we conducted direct comparisons of cell fractions for the landscape (Fig. 2c) as well as lineage subclusters across liver disease phases (Figs. 3d, 4d, 5c) and developmental stages (Fig. 6c). As shown in Fig. 6c, though fetal liver samples were combined from two datasets with different age range, frequencies of fetal Pre-B cells, DC cells and hepatocytes as expected demonstrated increasing tendency whereas that of hepatoblasts decreased by stage, agreeing with the source studies and previous findings.

**Fig. 8:**
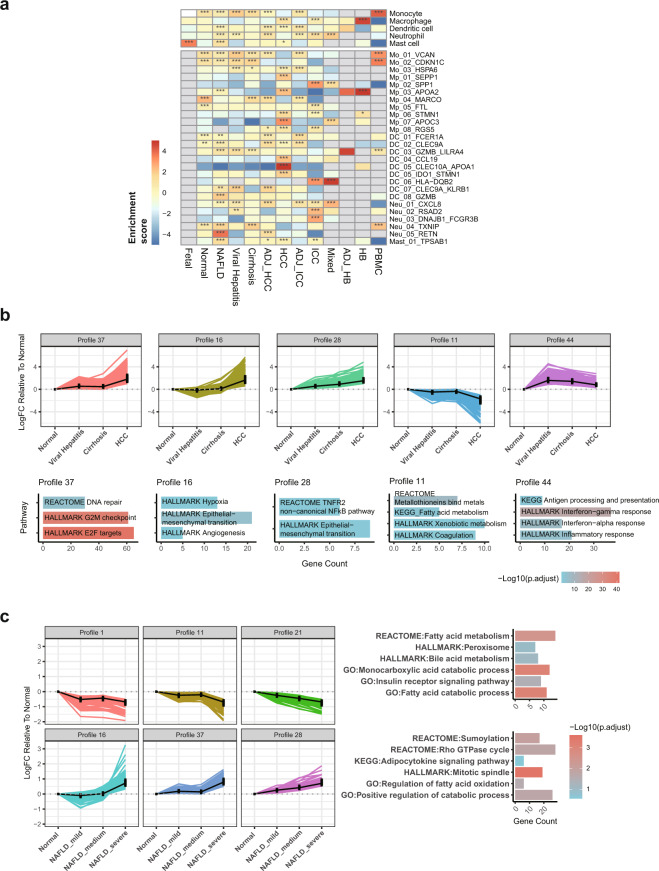
Interrogating the validity of dynamic changes over cell abundance and gene expression across liver phenotypes after data integration. (**a**) The enrichment OR value of myeloid cell types and subtypes evaluated across liver phenotypes based on relative cell fractions (Hypergeometric test, * for p < 0.05, ** for p < 0.01 and ***for p < 0.001); (**b**) Five typical expression patterns of mRNAs and lncRNAs along virus-associated liver disease course enriched with biological pathways involved in disease progression; (**c**) Six typical expression patterns of circRNAs were identified with the inflammation severity of NAFLD increasing. Pathways associated with lipid metabolism were overrepresented in host genes of both upregulated and downregulated circRNA clusters. Mo, monocyte; Mp, macrophage; DC, dendritic cell; Neu, neutrophil.

We further estimated the enrichment scores for myeloid subtypes in each liver phenotype and visualized them (Fig. 8a). The strong enrichment of monocytes in PBMC samples and Kupffer cells in Normal phenotype was consistent with physiological distributions of myeloid cells whereas the pro-fibrogenic macrophage Mp_02_SPP1 in ICC and conventional DC featuring LAMP3 expression (DC_CCL19) exhibited significant concentration in ICC and HCC respectively as frequently discussed in literature^26,40,41^ (Fig. 8a). These results, at least to some extent, demonstrated that phenotype-specific cell composition preferences could still be prominent and biological reasonable after data integration.

### Validity of gene expression dynamics during liver transitions after integration

Bulk RNA-seq datasets were reanalyzed from raw reads and then combined without batch effect adjustment in GepLiver to minimize batch variables across datasets while ensure the conservation of biological variance as possible. Thus, it is important to evaluate whether comparisons of gene expression among samples sourcing from different studies reflect biologically meaningful transcriptomic dynamics. To achieve this, we performed time-dependent clustering via STEM software on two typical transitional trajectories of liver diseases (Normal-Viral Hepatitis-Cirrhosis-HCC; Normal- NAFLD with inflammation level ranging from mild, medium to severe) for linear transcripts (mRNAs and lncRNAs) (Fig. 8b) and circRNAs (Fig. 8c) respectively. Along the virus-associated liver disease course, we identified 18 significant mRNA and lncRNA clusters of which 5 typical ones were demonstrated (Fig. 8b). Genes enriched in cell cycle, epithelial-mesenchymal transition and angiogenesis pathways were upregulated throughout virus associated liver diseases whereas liver metabolism-related processes displayed stepwise decreases. Immune pathways surged in viral hepatitis agreeing with the inflammatory infiltration while later reduced in cirrhosis and HCC. These dynamic changes of gene modules were consistent with findings investigated in Bulk23 dataset (GSE148355) and the activation of proliferation, the reprogramming of immune escape as well as epithelial dedifferentiation all have been widely reported in the initiation and progression of HCC^4,42,43^. For circRNA modules found continuously upregulated or downregulated along with the metabolism-related hepatitis inflammation grade, their host genes were predominantly enriched in pathways associated with lipid metabolism (Fig. 8c), validating that reasonable transcriptomic changes could be potentially recovered from the integrated data resource.

## Usage Notes

### Data access and future development

The data associated with this manuscript at figshare^14^ was peer-reviewed in 2023, while GepLiver.org represents a dynamic data resource.

Processed expression matrices and harmonized metadata of all datasets involved were available at figshare^14^ as well as the “Download” page of GepLiver website (www.GepLiver.org/#/download). As an ongoing atlas project, GepLiver will continuously incorporate liver expression profiles of local cohorts as well as the latest studies published, especially those featuring novel data modalities (eg. Spatial transcriptomics) or disease models. The interactive web portal will also be kept upgraded accordingly. With these efforts, our dynamic and integrative liver expression atlas is expected to facilitate the timely exploration of liver transcriptomics from every aspect and accelerate transcriptome-based basic and clinical research in hepatology field.

## Data Availability

The software used for quality control and data processing of GepLiver are as follows. 1. FastQC version 0.11.9 2. Trimmomatic version 0.33 3. STAR version 2.5.3a 4. StringTie version 1.2.3 5. FeatureCounts version 1.6.3 6. CIRI2 version 2.0.6 7. CellRanger version 6.0.0 8. R version 4.1.2 9. Seurat version 4.1.0 10. UCell version 1.3.1 11. inferCNV version 1.10.1 12. Monocle version 2.22.0 13. CytoTRACE version 0.3.3 14. CellPhoneDB version 3.1.0 15. STEM (Short Time-series Expression Miner) version 1.3.13 Custom code used for data processing and technical validation was provided in File “Custom R Scripts” deposited at figshare^14^.
